# Regulation of Gli ciliary localization and Hedgehog signaling by the PY-NLS/karyopherin-β2 nuclear import system

**DOI:** 10.1371/journal.pbio.2002063

**Published:** 2017-08-04

**Authors:** Yuhong Han, Yue Xiong, Xuanming Shi, Jiang Wu, Yun Zhao, Jin Jiang

**Affiliations:** 1 Department of Molecular Biology, University of Texas Southwestern Medical Center at Dallas, Dallas, Texas, United States of America; 2 State Key Laboratory of Cell Biology, CAS Center for Excellence in Molecular Cell Science, Innovation Center for Cell Signaling Network, Institute of Biochemistry and Cell Biology, Shanghai Institute of Life Sciences, CAS, School of Life Science and Technology, ShanghaiTech University, Shanghai, China; 3 Department of Physiology, University of Texas Southwestern Medical Center at Dallas, Dallas, Texas, United States of America; 4 Department of Pharmacology, University of Texas Southwestern Medical Center at Dallas, Dallas, Texas, United States of America; University of Pittsburgh, United States of America

## Abstract

Hedgehog (Hh) signaling in vertebrates depends on primary cilia. Upon stimulation, Hh pathway components, including Gli transcription factors, accumulate at primary cilia to transduce the Hh signal, but the mechanisms underlying their ciliary targeting remains largely unknown. Here, we show that the PY-type nuclear localization signal (PY-NLS)/karyopherinβ2 (Kapβ2) nuclear import system regulates Gli ciliary localization and Hh pathway activation. Mutating the PY-NLS in Gli or knockdown of Kapβ2 diminished Gli ciliary localization. Kapβ2 is required for the formation of Gli activator (Gli^A^) in wild-type but not in *Sufu* mutant cells. Knockdown of Kapβ2 affected Hh signaling in zebrafish embryos, as well as in vitro cultured cerebellum granule neuron progenitors (CGNPs) and SmoM2-driven medulloblastoma cells. Furthermore, Kapβ2 depletion impaired the growth of cultured medulloblastoma cells, which was rescued by Gli overexpression. Interestingly, Kapβ2 is a transcriptional target of the Hh pathway, thus forming a positive feedback loop for Gli activation. Our study unravels the molecular mechanism and cellular machinery regulating Gli ciliary localization and identifies Kapβ2 as a critical regulator of the Hh pathway and a potential drug target for Hh-driven cancers.

## Author summary

The secreted Hedgehog (Hh) protein plays an evolutionarily conserved role in both embryonic development and adult tissue homeostasis. Malfunction of Hh signaling activity contributes to a wide range of human diseases, including birth defects and cancer. Hh signaling in vertebrates critically depends on the primary cilium, a microtubule-based plasma membrane protrusion present on the surface of most mammalian cells. Upon ligand stimulation, Hh pathway components, including the seven-transmembrane protein Smoothened (Smo) and Gli transcription factors, accumulate at primary cilia to transduce the Hh signal, but the mechanisms underlying their ciliary targeting are still poorly understood. Here, we discover that the PY-type nuclear localization signal (PY-NLS) and the nuclear import factor karyopherinβ2 (Kapβ2) regulate Gli ciliary localization and Hh pathway activity. Mutating the PY-NLS in Gli or knockdown of Kapβ2 diminished Gli ciliary localization without affecting Smo ciliary accumulation in response to Hh. Kapβ2 regulates the formation of the active form of Gli, which is required for proper Hh signaling in zebrafish embryos and cultured cerebellum granule neuron progenitors (CGNPs). Kapβ2 depletion impaired the growth of medulloblastoma cells driven by an oncogenic form of Smo. Finally, Kapβ2 is a transcriptional target of the Hh pathway, forming a positive feedback loop to promote Gli activation. Our study reveals the molecular mechanism underlying the regulation of Gli ciliary targeting and identifies Kapβ2 as a potential cancer drug target.

## Introduction

Cell–cell signaling often occurs in specialized subcellular compartments. One such cell signaling center is the primary cilium, which is a microtubule-based plasma membrane protrusion [1]. Primary cilia regulate many essential cellular processes, and their malfunction is attributed to numerous human disorders collectively called “ciliopathy” [2]. Recently, the primary cilium has been implicated in transducing extracellular signals, most notably, the Hedgehog (Hh) signal [1,3].

The Hh family of secreted proteins plays pivotal roles in both embryonic development and adult tissue homeostasis [4–6]. Deregulation of Hh signaling activity has been linked to numerous human diseases, including birth defects and cancer [5,7–9]. The Hh signal is transduced by the seven-transmembrane G-protein-coupled receptor (GPCR)-like protein Smoothened (Smo), leading to activation of the latent Gli family of Zn-finger transcription factors. Both Smo and Gli are localized to primary cilia in response to Hh stimulation [10–14]; however, the mechanisms that target Hh pathway components to the primary cilia have remained poorly understood. Although ciliary localization of Smo and Gli proteins correlates with Hh pathway activation, definitive proof that ciliary localization of these and other pathway components is required for Hh signal transduction is still lacking. Indeed, a recent study revealed that Smo could activate the Hh signaling pathway in the absence of ciliary accumulation under certain conditions [15].

A ciliary localization signal has been identified in Smo; however, similar ciliary localization signals were not found in the Gli proteins [10]. A previous study suggested that nuclear localization signal (NLS) can function as a ciliary targeting signal for the kinesin-2 motor kinesin family member (KIF) 17 [16]; however, a recent study showed that deletion of the canonical NLS in Gli2 did not affect its ciliary localization [17]. In a previous study, we identified a noncanonical NLS called PY-type nuclear localization signal (PY-NLS) that matches the consensus: basic/hydrophobic motif-X7~12-R/K/H-X2~5-PY/L[18], which is localized in the N-terminal region of the Gli family of transcription factors, including the *Drosophila* Gli homolog Cubitus interruptus (Ci) and vertebrate Gli1, Gli2, and Gli3 (S1 Fig) [19]. We found that the PY-NLS acts in conjunction with the canonical NLS (a bipartite NLS) localized in the Zn-finger domain to promote efficient Ci nuclear localization in *Drosophila* [19]. In the process of dissecting the function of PY-NLS and canonical NLS in regulating Gli proteins, we found that the canonical NLS in Gli plays a major role, whereas the PY-NLS a minor role in targeting Gli to the nucleus. Interestingly, mutating the PY-NLS but not the canonical NLS impaired Gli ciliary localization. The PY family of NLSs interacts with the karyopherin-β family member karyopherin-β2 (Kap-β2; also known as Transportin 1 or importin β2) that transports PY-NLS-containing proteins to the nucleus [18]. Kapβ2 is required for the ciliary localization of retinitis pigmentosa 2 (RP2) [20]. Here, we show that Kap-β2 is essential for Gli ciliary localization and activation. Inactivation of Kap-β2 inhibited Hh signal transduction in cultured mammalian cells, as well as in zebrafish embryos. Furthermore, Kap-β2 is essential for the growth of cultured cerebellum granule neuron precursors (CGNPs) as well as medulloblastoma cells driven by a *smo* oncogenic mutation (*SmoM2*). Interestingly, we find that Kap-β2 itself is a target of the Hh–Gli signaling pathway, suggesting that Kap-β2 and Hh–Gli forms a positive feedback loop to promote Gli ciliary localization and activation.

## Results

### PY-NLS is required for efficient Gli ciliary localization

**Fig 1:**
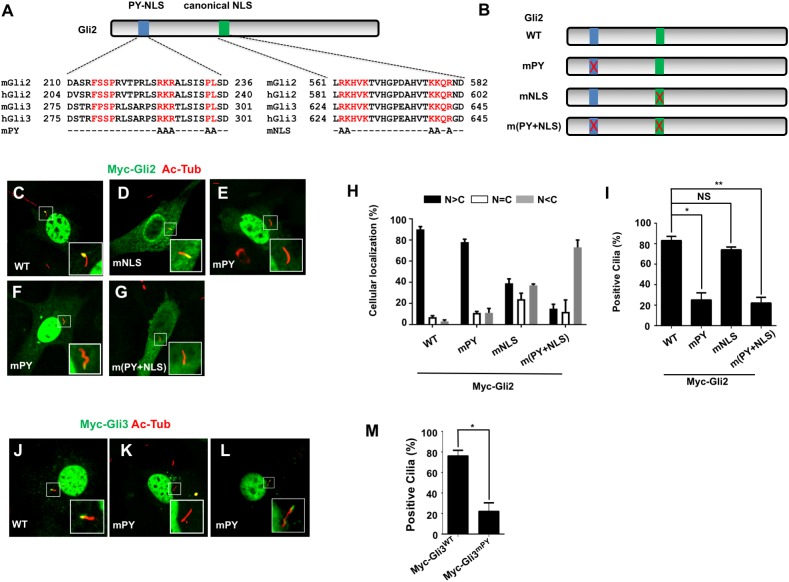
The PY-type nuclear localization signal (PY-NLS) is required for Gli ciliary localization. (**A**) Diagram of mGli2 with its PY-NLS and canonical nuclear localization signal (NLS) indicated by the blue and green boxes, respectively. Sequence alignment of the PY-NLS and canonical NLS is shown underneath. (**B**) Diagram of wild-type mGli2 and its mutant forms. (**C–G**, **J–I**) Subcellular localization of Myc-tagged wild type Gli2 (**C**), Gli2^mNLS^ (**D**), Gli2^mPY^ (**E–F**), Gli2^m(PY+NLS)^ (**G**), wild-type Gli3 (**J**), or Gli3^mPY^ (**K–I**) transiently expressed in NIH3T3 cells. Acetylated tubulin (Ac-Tub) marks primary cilia. Insets show the enlarged views of the indicated areas. (**H**) Nuclear versus cytoplasmic localization of the indicated Gli2 variants transfected into NIH3T3 cells (50 cells were counted for each Gli construct). N > C: nuclear signal intensity is stronger than that of cytoplasmic signal. N = C: nuclear signal intensity is comparable to that of cytoplasmic signal. N < C: nuclear signal intensity is weaker than that of cytoplasmic signal. (**I**, **M**) Quantitation of the ciliary localization of wild-type and mutant Myc-Gli2 (**I**) or Myc-Gli3 (**M**). *N* = 100 cells were examined for each Gli construct. Data are means ± SD from 2 independent experiments. *P < 0.*05*, **P < 0.*01*, NS: not significant. The underlying data for this figure can be found in S1 Data.

To study the function of the PY-NLS in the Gli proteins, we generated full-length Gli2 and Gli3 lacking the PY-NLS (Gli2^mPY^ and Gli3^mPY^), as well as full-length Gli2 lacking the canonical NLS (Gli2^mNLS^) or lacking both the PY and canonical NLS (Gli2^m(PY+NLS)^) (Fig 1A and 1B). When transfected into NIH3T3 cells, Myc-tagged wild type Gli2 (Myc-Gli2^WT^) localized predominantly to the nucleus (Fig 1C and 1H) and the majority of transfected cells (> 80%) contained ciliary Myc-Gli2^WT^ signal (Fig 1C and 1I). Mutating the canonical NLS (Gli2^mNLS^) greatly reduced Gli2 nuclear localization but did not affect its ciliary localization (Fig 1D and 1H). By contrast, mutating the PY-NLS (Myc-Gli2^mPY^) only slightly reduced Gli2 nuclear localization but greatly impaired ciliary localization of Gli2, as only about 20% of transfected cells contained weak Myc-Gli2^mPY^ signal in primary cilia (Fig 1E, 1F, 1H and 1I). Although Gli2^m(PY+NLS)^ exhibited a more profound defect in its nuclear localization, its ciliary localization defect was similar to that of Myc-Gli2^mPY^ (Fig 1G–1I). Furthermore, Gli3^mPY^ exhibited diminished ciliary localization similarly to Gli2^mPY^ (Fig 1J–1M). These observations suggest that the PY-NLS in Gli proteins has acquired a new function, i.e., ciliary targeting of Gli, beyond its traditional role as a NLS.

### The PY-NLS is required for Gli2 activation

**Fig 2:**
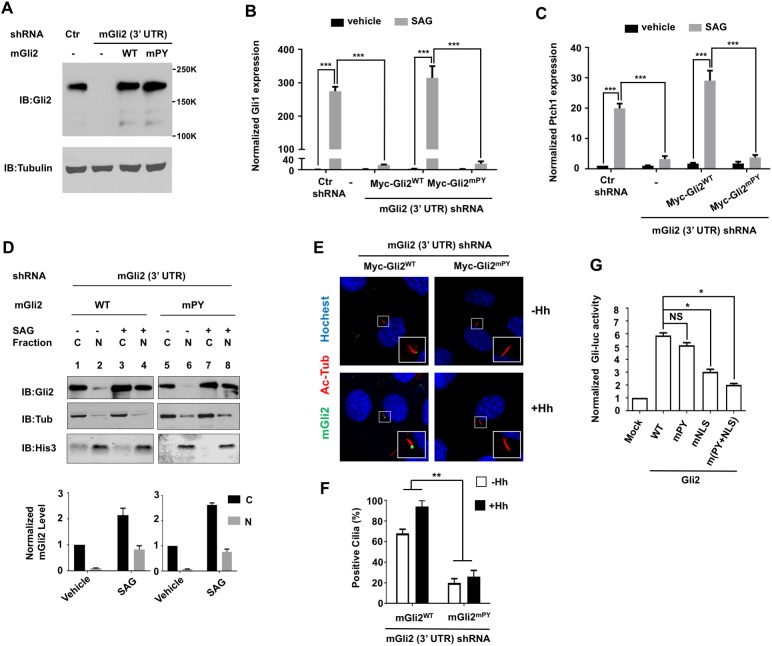
The PY-type nuclear localization signal (PY-NLS) is required for Gli activation. (**A**) Western blot of cell lysates from the indicated cell lines indicated that Myc-Gli2^WT^ and Myc-Gli2^mPY^ were expressed at comparable levels that were slightly higher than that of endogenous Gli2. (**B–C**) Normalized mRNA levels of endogenous *Gli1* (**B**) or *Patch1* (**C**) measured by quantitative reverse transcription PCR (RT-qPCR) in control (green fluorescent protein [GFP] short hairpin RNA [shRNA]) or Gli2-depeleted NIH3T3 cells with or without lentiviral infection of the indicated Gli2 constructs. (**D**) Fractionation of Myc-Gli2^WT^ and Myc-Gli2^mPY^ from the indicated cell lines treated with or without SAG. Quantification of protein level is shown in the bottom panel. (**E–F**) Immunostaining (**E**) and quantification (**F**) of ciliary-localized Myc-Gli2^WT^ or Myc-Gli2^mPY^ in NIH3T3^mGli2-shRNA^ cells treated with or without sonic hedgehog (Shh). *N* = 50 cells were examined for each Gli construct. Data are means ± SD from 2 independent experiments. **P < 0.*01*. (**G**) *Gli-luciferase* assay was performed in NIH3T3 cells transfected with the indicated constructs. Data are means ± SD from 2 independent experiments. *P < 0.05, NS: not significant. The underlying data for this figure can be found in S1 Data.

It has been proposed that ciliary translocation of Hh pathway components, including Gli proteins, is essential for Hh signal transduction leading to Gli activation [1, 3]. However, many studies addressing the importance of ciliary localization of Gli in Hh signal transduction involved disruption of cilia structure or components involved in ciliary protein transport [21] [12, 13, 22]; as such, alternative explanation cannot be excluded. The ciliary localization defect associated with Gli2^mPY^ provided us an opportunity to directly test whether ciliary localization of Gli2 is essential for its activation. To this end, we established NIH3T3 cell lines with endogenous Gli2 depleted by RNA interference (RNAi) targeting its 3′ untranslated region (NIH3T3^mGli2-shRNA^) and supplemented with or without the expression of exogenous Myc-Gli2^WT^ or Myc-Gli2^mPY^ at low levels using the lentiviral transfection system. Western blot analysis indicated that Myc-Gli2^WT^ and Myc-Gli2^mPY^ were expressed at levels slightly higher than that of the endogenous Gli2 (Fig 2A). We found that Gli2 depletion diminished the expression of both *Ptch1* and *Gli1*, two sonic hedgehog (Shh) target genes, in response to SAG (Fig 2B and 2C). SAG-stimulated expression of *Ptch1* and *Gli1* in NIH3T3^mGli2-shRNA^ cells was fully restored by the expression of Myc-Gli2^WT^ (Fig 2B and 2C). By contrast, Myc-Gli2^mPY^ only marginally restored the expression Shh target genes in NIH3T3^mGli2-shRNA^ cells and exhibited approximately 20-fold less activity compared to Myc-Gli2^WT^ (Fig 2B and 2C).

Fractionation experiments indicated that Myc-Gli2^mPY^ only exhibited a slight reduction in its nuclear localization compared with Myc-Gli2^WT^ (Fig 2D), which cannot account for the large drop in Myc-Gli2^mPY^ activity. On the other hand, ciliary localization of Myc-Gli2^mPY^ was greatly diminished under both unstimulated and Hh-stimulated conditions compared to Myc-Gli2^WT^ (Fig 2E and 2F), which correlated with its diminished activity. In transient transfection experiments where both Myc-Gli2^WT^ and Myc-Gli2^mPY^ were overexpressed, Myc-Gli2^mPY^ activated a *Gli-luciferase* (*Gli-luc*) reporter gene similar to Myc-Gli2^WT^ (Fig 2G), consistent with a previous finding that an excessive amount of Gli2 can activate Hh pathway independent of the primary cilia [13]. On the other hand, both Myc-Gli2^mNLS^ and Myc-Gli2^m(PY+NLS)^ exhibited much-reduced ability in activating the *Gli-luc* reporter gene (Fig 2G), consistent with their intrinsic nuclear localization defect (Fig 1H). These observations suggest that the defect in Hh signaling activity associated with Myc-Gli2^mPY^ is most likely due to its ciliary localization defect. Hence, the PY-NLS–mediated ciliary localization of Gli2 is required for its activation in response to the upstream signal. These results are consistent with a recent study showing that a nonciliary Gli2 deletion mutant failed to respond to Hh when knocked into the endogenous locus [23].

### The PY-NLS is not sufficient for ciliary targeting

**Fig 3:**
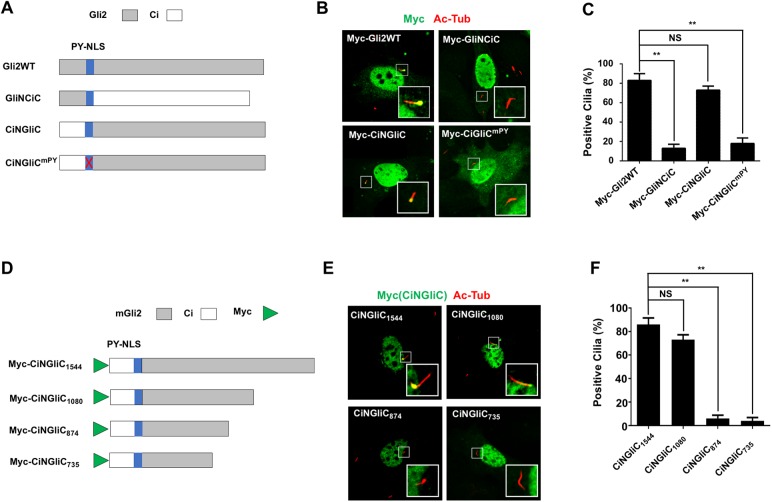
The C-terminal region of Gli2 is required for its ciliary localization. (**A**) Diagram of wild-type mGli2 and Gli2/Ci chimeric proteins. (**B–C**) Ciliary localization (**B**) and its quantification (**C**) of the indicated Gli2/Ci constructs. Data are means ± SD from 2 independent experiments. **P < 0.01, NS: not significant. (**D**) Diagram of the Gli2/Ci chimeric protein and its C-terminal deletion constructs. (**E–F**) Ciliary localization (**E**) and its quantification (**F**) of Gli2/Ci and its deletion mutants. Data are means ± SD from 2 independent experiments. **P < 0.01, NS: not significant. The underlying data for this figure can be found in S1 Data.

We have previously shown that the PY-NLS motif from Gli2, Gli3, or Ci, when fused to a heterologous protein such as LacZ, is sufficient to confer nuclear translocation of the heterologous protein (S1A and S1B Fig) [19]. However, the PY-NLS was unable to confer ciliary localization when fused to LacZ (S1B Fig). Consistent with the notion that the PY-NLS is insufficient for ciliary targeting, Ci was not localized to the primary cilium when expressed in NIH3T3 cells. To determine additional domain(s) in Gli2 required for its ciliary localization, we generated several Ci-Gli2 chimeric proteins (Fig 3A). Replacing the Gli2 sequence C-terminal to the PY-NLS motif with that of Ci (GliNCiC) nearly abolished the ciliary localization of the chimeric protein (Fig 3B and 3C). On the other hand, replacing the Ci sequence C-terminal to the PY-NLS motif with that of Gli2 (CiNGliC) conferred ciliary localization of the chimeric protein, and mutating the PY-NLS in CiNGliC (CiNGliC^mPY^) diminished its ciliary localization (Fig 3A–3C). These results suggest that the PY-NLS in Ci is a functional ciliary targeting signal but the Gli sequence C-terminal to its PY-NLS is also required for Gli ciliary localization.

To narrow down the C-terminal domain required for Gli ciliary localization, we generated a set of C-terminally deleted CiNGliC variants (Fig 3D). Deletion to amino acid (aa) 1080 (CiN-GliC1080) reduced, whereas deletion to aa 874 (CiN-GliC874) or aa 735 (CiN-GliC735) abolished ciliary localization (Fig 3E and 3F), suggesting the sequence between aa 874 and aa 1080 of Gli2 is critical for its ciliary localization. Consistent with this, 2 recent studies also identified sequence overlapping with this region as critical for Gli2 ciliary localization [17, 23].

### Kapβ2 mediates ciliary translocation of Gli proteins

**Fig 4:**
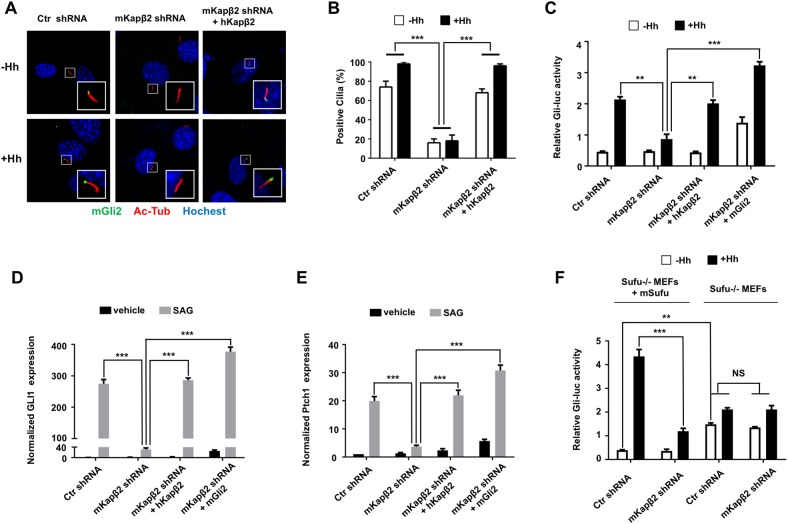
Karyopherinβ2 (Kapβ2) is required for Gli ciliary location and Hedgehog (Hh) pathway activation. (**A–B**) Ciliary localization of endogenous Gli2 in NIH3T3 cells infected with lentivirus containing short hairpin RNA (shRNA) targeting either green fluorescent protein (GFP) (Ctr) or mouse karyopherinβ2 (mKapβ2) (with or without reintroducing human karyopherinβ2 [hKapβ2] by a second round of lentiviral infection) and treated with or without sonic hedgehog (Shh). Quantitation of Gli2 ciliary localization is shown in (**B**). One hundred cells were examined for each condition. Data are means ± SD from 2 independent experiments. ***P < 0.001. (**C–E**) *Gli-luc* reporter assay (**C**), *Gli1* (**D**), or *Ptch1* (**E**) expression in NIH3T3 cells infected with lentivirus expressing GFP (Ctr) shRNA or mKapβ2 shRNA with or without hKapβ2 or mGli2 coexpression. Cells were treated with or without sonic hedgehog N-terminal fragment (Shh-N) (**C**) or SAG (**D–E**) as indicated. (**F**) *Gli-luc* reporter assay in the indicated cell lines treated with or without Shh. Data are means ± SD from 2 independent experiments. **P < 0.01, ***P < 0.001, NS: not significant. The underlying data for this figure can be found in S1 Data.

The PY family of NLSs physically interacts with Kapβ2, which carries the PY-NLS–containing cargoes into the nucleus [18]. Indeed, an N-terminal fragment of Ci, Ci1-440 (CiN), interacted with the *Drosophila* Kapβ2, Trn, in a manner depending on the PY-NLS, and depletion of Trn diminished CiN nuclear localization in S2 cells [19]. To determine whether Kapβ2 is required for ciliary localization of Gli proteins, we depleted mouse karyopherinβ2 (mKapβ2) from NIH3T3 by establishing cell lines stably expressing 2 independent short hairpin RNAs (shRNAs) (mKapβ2-shRNA1 and mKapβ2-shRNA2) that targeted different regions of mKapβ2. We found that both mKapβ2-shRNA1 and mKapβ2-shRNA2 effectively knocked down endogenous mKapβ2 and diminished ciliary localization of endogenous Gli2, both in the absence and in the presence of Hh stimulation (Fig 4A and 4B; S2A and S2B Fig). Because mKapβ2-shRNA2 knocked down mKapβ2 with a higher efficiency than mKapβ2-shRNA1 (S3A Fig), we focused on this RNAi line for the rest of the study and simply referred it to as mKapβ2-shRNA unless mentioned otherwise. The ciliary localization defect of Gli2 in mKapβ2-shRNA–expressing cells was completely rescued by transfection with a human karyopherinβ2 (hKapβ2) that is resistant to mKapβ2-shRNA (Fig 4A and 4B). mKapβ2-shRNA also diminished ciliary-localized Myc-Gli3 and Flag-Gli1, and this defect was completely rescued by coexpressing hKapβ2 (S2C–S2F Fig). The observations that 2 independent mKapβ2-shRNAs can both inhibit Gli ciliary localization and that such defect can be rescued by hKapβ2 rule out off-target effect and demonstrate that Kapβ2 is essential for ciliary localization of Gli proteins.

To determine whether Kapβ2 is required for Smo ciliary translocation, NIH3T3 cells expressing mKapβ2-shRNA or green fluorescent protein (GFP)-shRNA were infected with lentivirus expressing a Myc-tagged form of Smo (Myc-Smo) and treated with or without Hh. We found that Hh stimulated ciliary accumulation of Myc-Smo in both control and mKapβ2 depleted cells (S3A and S3B Fig), suggesting that Kapβ2 is not required for Smo ciliary localization.

In addition, ciliary localization of a yellow fluorescent protein (YFP)-tagged KIF7, a Hh pathway component that is also required for cilium tip organization [24–27], was not affected by Kapβ2 knockdown (S3C Fig). Taken together, these observations suggest that the ciliary localization defect of Gli proteins caused by Kapβ2 knockdown is not likely due to a general defect in ciliary structure and/or transport but rather to a specific role of Kapβ2 in the regulation of Gli ciliary localization.

### Kapβ2 is essential for Hh pathway activation

The observation that Kapβ2 is required for Gli ciliary localization prompted us to examine whether depletion of Kapβ2 affects Hh target gene expression. We found that Kapβ2 RNAi diminished the *Gli-luc* reporter activity, as well as the expression of endogenous *Gli1* and *Ptch1* induced by Hh, and that this defect was fully rescued by the expression of hKapβ2 (Fig 4C–4E). In addition, introducing Myc-Gli2 into mKapβ2-shRNA cells by lentiviral infection restored the expression of Hh target genes (Fig 4C–4E), suggesting that down-regulation of Hh target genes in Kapβ2-depleted cells is due to diminished Gli activator activity.

We then examined the relationship between Kapβ2 and Sufu, a major negative regulator of the mammalian Hh pathway that binds and inhibits Gli proteins [13, 28]. Previous studies suggest that the constitutive Gli activity in Sufu mutant cells is cilium-independent [13, 29]. We reasoned that if Kapβ2 promotes Hh pathway activity by targeting Gli proteins to primary cilia, then removing Sufu should bypass the requirement of Kapβ2 for Hh pathway activation. To test this, we depleted mKapβ2 from *Sufu*^*-/-*^ mouse embryonic fibroblast (MEF) cells by viral infection of mKapβ2-shRNA. As a control, we reintroduced mSufu into *Sufu*^*-/-*^ MEF cells to generate *Sufu*^*+*^ MEFs (*Sufu*^*-/-*^ + *mSufu*). We found that Kapβ2 RNAi diminished Hh-induced Gli-luc activity in *Sufu*^*+*^ MEF cells (Fig 4F); however, the high basal as well as Hh-induced Gli-luc activity was not affected by Kapβ2 depletion in *Sufu*^*-/-*^ MEFs (Fig 4F), suggesting that Kapβ2 is not required for the ectopic Gli activity in the *Sufu* mutant background. Of note, Hh-induced *Gli-luc* activity was relatively low in *Sufu*^*-/-*^ MEFs compared with *Sufu*^*+*^ MEFs because Gli proteins are unstable in the absence of Sufu[13]. The observation that the Hh signaling defect caused by Kapβ2 depletion was fully rescued by overexpression of Gli2 or removal of Sufu strongly implies that the signaling defect is mainly due to lack of Gli activation rather than a general defect caused by Kapβ2 inactivation.

### Kapβ2 is required for Hh signaling in zebrafish embryos

**Fig 5:**
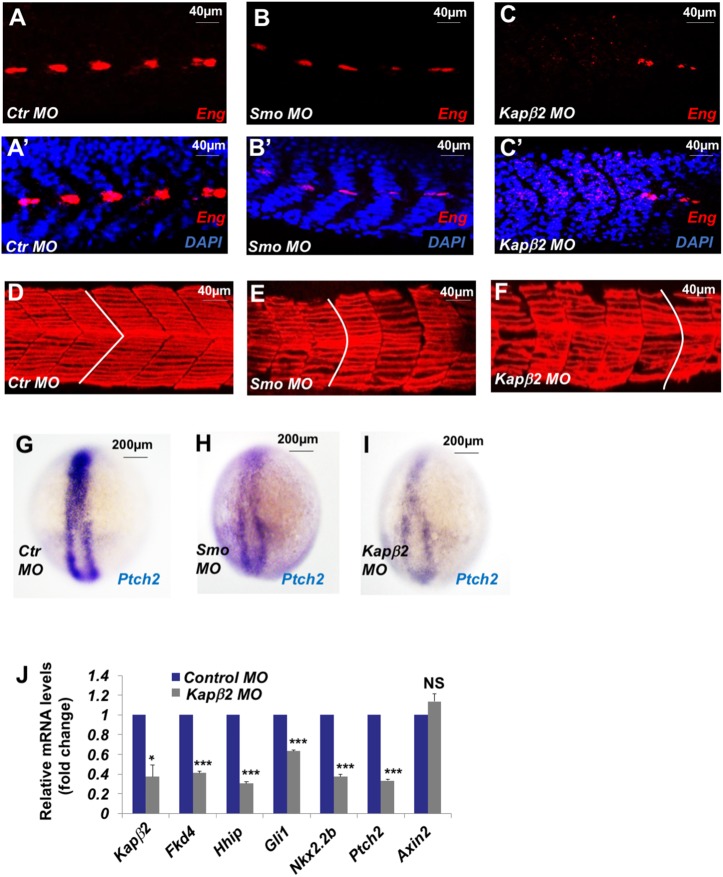
Karyopherinβ2 (Kapβ2) regulates Hedgehog (Hh) signaling in zebrafish embryos. (**A–C′**) Zebrafish embryos injected with the indicated morpholinos (MOs) were immunostained at 24 hours post-fixation (hpf) with Engrailed (Eng) antibody (red) and DAPI (blue) to visualize nuclei. (**D–F**) Zebrafish embryos injected with the indicated MOs were immunostained at 24 hpf with F59 antibody (red) to visualize slow muscle fibers. (**G–I**) Zebrafish embryos injected with the indicated MOs were analyzed for Ptch2 expression at 10 hpf by in situ hybridization. (**J**) Relative mRNA levels of the indicated genes from 24 hpf zebrafish embryos injected with the indicated MOs were measured by quantitative reverse transcription PCR (RT-qPCR). Data are means ± SD from 3 independent experiments. *P < 0.05, ***P < 0.001, NS: not significant. The underlying data for this figure can be found in S1 Data.

To determine whether Kapβ2 regulates Hh pathway in vivo, we turned to zebrafish and inactivated Kapβ2 during embryonic development by injecting morpholinos (MOs) into 1-cell stage embryos (see Materials and methods). We found that Kapβ2 MO resulted in reduced expression of the Hh target gene *Eng* and “U-shaped” somites similar to Smo MO (Fig 5A–5F), phenotypes indicative of Hh signaling defects[30]. In addition, Kapβ2 MO led to reduced expression of multiple Hh target genes, including *Ptch2*, *Hhip*, *Nkx2*.*2b*, and *Gli1* as determined by in situ hybridization and/or real-time PCR (Fig 5G–5J). Under these circumstances, Kapβ2 MO did not affect the expression of the Wnt target gene *Axin2* (Fig 3J), consistent with a previous report that the primary cilium is not required for Wnt signaling in zebrafish [31].

### Kapβ2 is required for cultured CGNP proliferation and medulloblastoma growth

**Fig 6:**
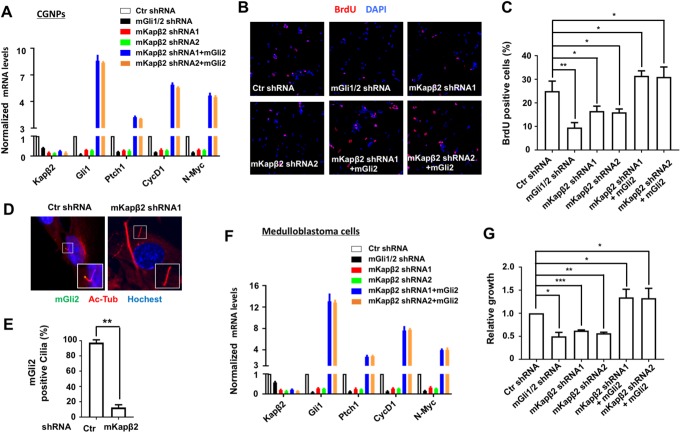
Karyopherinβ2 (Kapβ2) regulates cell growth of cultured cerebellum granule neuron precursors (CGNPs) and medulloblastoma. (**A**) Relative mRNA levels of the indicated genes in cultured CGNPs infected with lentiviruses expressing control, mGli1/2, and mouse karyopherinβ2 (mKapβ2) short hairpin RNA (shRNA) with or without mGli2 coexpression were measured by quantitative reverse transcription PCR (RT-qPCR) (means ± SD, *n* = 3). (**B–C**) Immunostaining and quantification of bromodeoxyuridine (BrdU) incorporation in cultured CGNPs expressing the indicated shRNAs and transgenes (means ± SD, *n* = 3). *P < 0.05, **P < 0.01. (**D–E**) Immunostaining (**D**) and quantification (**E**) of ciliary-localized Gli2 in control or mKapβ2 knockdown CGNPs treated with sonic hedgehog (Shh). *N* = 50 cells were examined for each condition. Data are means ± SD from 2 independent experiments. **P < 0.*01*. (**F**) Relative mRNA levels of the indicated genes in cultured SmoM2-driven medulloblastoma cells infected with lentiviruses expressing control, mGli1/2, and mKapβ2 shRNA with or without mGli2 coexpression were measured by RT-qPCR (means ± SD, *n* = 3). (**G**) Cultured SmoM2-driven medulloblastoma cells expressing the indicated shRNAs with or without mGli2 were subjected to the ATP cell viability assay, and the relative survival rates were indicated (means ± SD, *n* = 3). *P < 0.05, **P < 0.01, ***P < 0.001. The underlying data for this figure can be found in S1 Data.

During cerebellum development from the late embryonic stage to the early postnatal stage, Hh signaling is required for the proliferation and expansion of the CGNPs in the external granule layer (EGL) [32–34]. To determine whether Kapβ2 regulates Shh signaling in CGNPs that are essential for their proliferation, we inactivated Kapβ2 using RNAi (mKapβ2 shRNA1 or mKapβ2 shRNAi2) in Shh-treated mouse CGNP cultures. We also depleted both mouse Gli1 and Gli2 by RNAi (mGli1/2 shRNA) in Shh-treated CGNP cultures in parallel experiments. We found that knockdown of Kapβ2 in CGNPs significantly impaired the expression of Shh target genes such as *Gli1*, *Ptch1*, *Cyclin D1* (*CycD1*), and *N-Myc* and inhibited the proliferation of CGNPs as determined by bromodeoxyuridine (BrdU) incorporation in a manner similar to Gli1/2 depletion (Fig 6A–6C) [35]. Introducing exogenous mGli2 into Kapβ2-depleted CGNPs restored Shh target gene expression above the basal levels and rescued the proliferation defect of Kapβ2 depleted CGNPs (Fig 6A–6C). Consistent with Kapβ2 regulating Shh pathway activity through Gli ciliary localization, ciliary localization of endogenous Gli2 was diminished in Kapβ2-depleted mouse CGNPs (Fig 6D–6E).

Mutations leading to constitutively active Shh signaling cause Shh-subtype medulloblastoma, whose progression requires the active pathway activity [36–39]. Therefore, we determined whether Kapβ2 is required for the growth of medulloblastoma driven by SmoM2, which resulted in constitutive activation of Smo [37, 40]. SmoM2-induced medulloblastoma cells were cultured in vitro for a short period of time and infected with lentiviruses expressing shRNAs for Kapβ2 or Gli1/2 [35]. Similar to Gli1/2 RNAi, Kapβ2 knockdown in SmoM2-induced medulloblastoma cells diminished the expression of Shh target genes, including *Gli1*, *Ptch1*, *CycD1*, and *N-Myc* (Fig 6F), leading to growth inhibition of the medulloblastoma cells as indicated by a cell survival assay (Fig 6G). Moreover, inhibition of Shh target gene expression and medulloblastoma growth were rescued by lentiviral infection of mGli2 into Kapβ2-depleted medulloblastoma cells (Fig 6F–6G). Taken together, these results suggest that Kapβ2-meidated Gli activation is required for Shh-stimulated CGNP proliferation and SmoM2-driven medulloblastoma cell growth.

### Kapβ2 forms a positive feedback loop to regulate the formation of Gli activator

**Fig 7:**
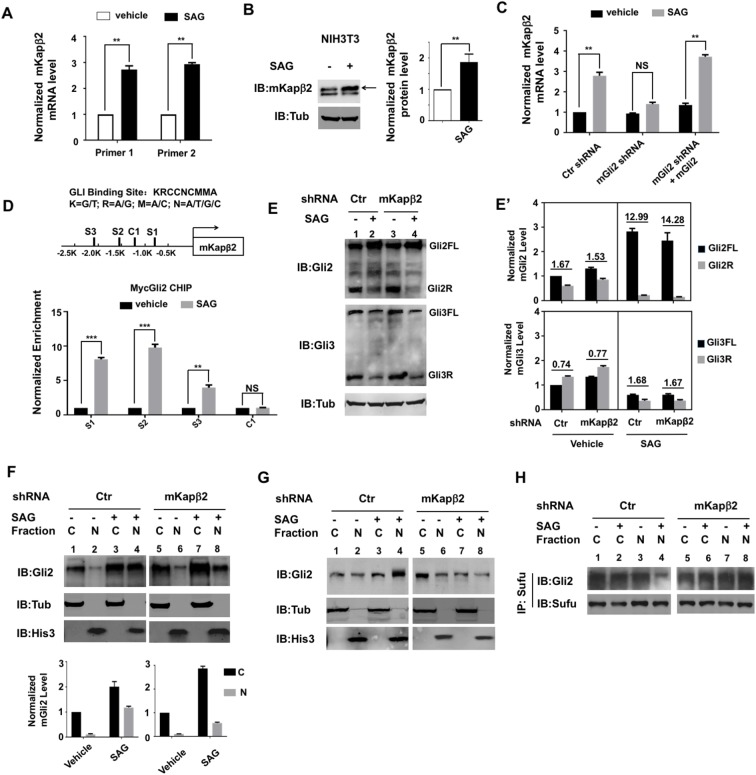
Karyopherinβ2 (Kapβ2) forms a positive feedback loop to regulate Gli activation. (**A–B**) Mouse karyopherinβ2 (mKapβ2) mRNA level (**A**) and protein abundance in NIH3T3 cells treated with or without SAG. Data are means ± SD from 2 independent experiments. **P < 0.01. Arrow in (**B**) indicates mKapβ2. (**C**) Gli2 depletion blocked SAG-induced mKapβ2 up-regulation. Data are means ± SD from 2 independent experiments. ** *P* < 0.01. (**D**) Top, diagram of the *mKapβ2* promoter region with 3 Gli binding consensus sites (S1 to S3) and a control region (C1) indicated. Bottom, Myc-Gli2 chromatin immunoprecipitation (CHIP) assay indicates increased occupancy of Gli2 to the 3 binding sites but not the control region in response to SAG. Data are means ± SD from 2 independent experiments. **P < 0.01, ***P < 0.001. (**E–E′**) Western blot analysis of Gli processing from control or mKapβ2-depleted NIH3T3 cells treated with or without SAG. Protein abundance of Gli^FL^ and Gli^R^ were quantified and their ratios indicated by the underlined numbers (**E′**). Data are means ± SD from 2 independent experiments. (**F**) Western blot analysis of cytoplasmic (C) and nuclear (N) fractions of endogenous Gli2 from control or mKapβ2-depleted NIH2T3 cells treated with or without SAG. Quantification of protein level is shown in the bottom panel. (**G**) Western blot analysis of cytoplasmic and nuclear fractions of endogenous Gli2 from control or mKapβ2-depleted NIH2T3 cells treated with or without SAG. Of note, cytoplasmic fractions were under-loaded to achieve similar intensity of individual bands. SAG-induced mobility shift of nuclear Gli2 was abolished by mKapβ2 short hairpin RNA (shRNA). (**H**) Cytoplasmic and nuclear fractions of cell lysates from control or mKapβ2-depleted NIH2T3 cells treated with or without SAG were immunoprecipitated with Sufu antibody, followed by western blot analysis. The underlying data for this figure can be found in S1 Data.

We noticed that Kapβ2 was up-regulated in the mouse model of medulloblastoma driven by SmoM2 (S4A Fig). In addition, Kapβ2 expression level is significantly higher in the Shh subgroup of medulloblastoma compared with the Wnt subgroup of medulloblastoma from clinical samples (S4B Fig). We found that depletion of Gli1/2 from Shh-stimulated CGNPs or SmoM2-driven medulloblastoma cells (Smo MO) in zebrafish embryos down-regulated the expression of Kapβ2 (Fig 6A and 6F, S4C Fig), suggesting that *Kapβ2* is a Shh-responsive gene. As a further support to this notion, NIH3T3 cells treated with SAG exhibited elevated *Kapβ2* mRNA levels and protein abundance (Fig 7A and 7B). Depletion of mGli2 from NIH3T3 cells abolished SAG-stimulated Kapβ2 up-regulation, which was rescued by lentiviral infection of exogenous mGli2 (Fig 7C), suggesting that Smo activation induces Kapβ2 expression through Gli.

Inspection of the *mKapβ2* gene locus identified 3 Gli protein binding consensus sites within a 2.5-kb sequence upstream from the transcription start site (Fig 7D). Furthermore, DNA fragments containing these sites were enriched in Myc-Gli2 chromatin immunoprecipitation (CHIP) in response to SAG stimulation (Fig 7D), suggesting that *mKapβ2* is a Gli target gene.

The primary cilium is required for the formation of both Gli^R^ and Gli^A^ [12, 21]; however, we found that Kapβ2 depletion in NIH3T3 cells did not significantly affect the proteolytic processing of either Gli2 or Gli3 to generate Gli^R^ (Fig 7E and 7E′). Furthermore, the ability of SAG to inhibit Gli^R^ formation was not affected by Kapβ2 depletion (Fig 7E and 7E′). These results imply that ciliary localization of Gli2/3 might not be absolutely required for their processing. Therefore, the Shh signaling deficiency caused by Kapβ2 depletion is most likely due to a defect in the conversion of Gli^FL^ to Gli^A^.

Previous studies suggested that Shh stimulates nuclear translocation of Gli2^FL^ [21] and that nuclear Gli2^FL^ exhibited increased phosphorylation and decreased association with Sufu [41–43], all of which may contribute to Gli2 activation. Consistent with Kapβ2 regulating Gli2 activation, we found that Kapβ2 depletion in NIH3T3 cells attenuated SAG-induced nuclear translocation of endogenous Gli2^FL^ (Fig 7F) abolished SAG-stimulated phosphorylation of nuclear Gli2^FL^ as indicated by the mobility shift on SDS-PAGE (Fig 7G). In control cells, SAG induced dissociation of Gli2^FL^ from Sufu in the nuclear fraction as measured by co-immunoprecipitation assay (Fig 7H, lanes 3–4)[42]; however, such dissociation was diminished in Kapβ2-depleted cells (Fig 7H, lanes 7–8). The sustained binding of Sufu to Gli2^FL^ in Kapβ2-depleted cells may explain why Gli2 cannot be activated in response to Shh in these cells.

## Discussion

**Fig 8:**
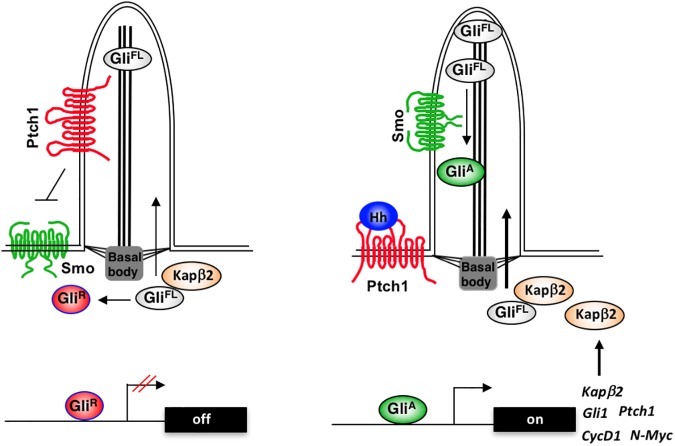
Karyopherinβ2 (Kapβ2) regulates Gli ciliary localization and activation. Kapβ2 binds the PY-type nuclear localization signal (PY-NLS) in Gli to promote its ciliary localization. In the absence of Hedgehog (Hh), Patched (Ptc) inhibits Smoothened (Smo), allowing Gli to be processed into Gli^R^ that translocates to the nucleus to inhibit Hh target gene expression. Hh binding to Ptc leads to ciliary accumulation of the activated form of Smo, which converts ciliary-localized Gli into Gli activator (Gli^A^) that translocates to the nucleus to activate the expression of Hh target genes, including Kapβ2. Kapβ2 up-regulation may further promote ciliary localization and activation of Gli.

Although it has been long thought that ciliary localization of Gli is essential for its activation and subsequent translocation into the nucleus, how Gli proteins are targeted to the primary cilia has remained a mystery. In this study, we identified the PY-NLS located in the N-terminal region of Gli proteins as a ciliary localization signal (CLS) whose mutation diminished Gli ciliary localization. We found that Kapβ2, which normally brings PY–NLS-containing cargoes into the nucleus, is essential for Gli ciliary localization and activation. Interestingly, *Kapβ2* itself is a Gli target gene, suggesting a positive feedback regulation of Gli activation (Fig 8). We provided further evidence that Kapβ2-mediated ciliary localization of Gli is essential for Hh pathway activity in multiple physiologically relevant contexts and depletion of Kapβ2 affected the growth of SmoM2-driven medulloblastoma cells cultured in vitro, suggesting that blockage of Kapβ2-mediated Gli ciliary localization may serve as a new strategy to treat Hh-driven cancers such as basal cell carcinoma (BCC) and medulloblastoma.

Members of the Gli family of transcription factors, including *Drosophila* Ci, contain a conserved PY-NLS in their N-terminal region and a canonical bipartite NLS in their Zn-finger DNA-binding domains. Whereas both NLSs contribute to the regulation of Ci nuclear localization [19], the PY-NLS has only a minor role in Gli nuclear targeting but instead plays a critical role in Gli ciliary localization (Fig 1). Hence, the PY-NLS has been co-opted by the Gli transcription factors for their ciliary targeting. Consistent with our findings that the PY-NLS/ Kapβ2 nuclear transport system regulates Gli ciliary localization and Hh pathway activity, a recent study revealed that blocking Kapβ2/Imp-β2 activity using a blocking peptide, M9M, also attenuated Gli2 ciliary localization without apparently affecting cilia length [44]. However, these authors failed to reveal a role of the PY-NLS in Gli2 ciliary localization, likely because the PY-NLS motif was insufficiently mutated in their study [44]. Several other studies attempted to map the ciliary localization signals for Gli proteins [17, 45]. Consistent with the PY-NLS in Gli ciliary targeting, several N-terminal deletion mutants with the PY-NLS motif removed exhibited significantly reduced ciliary localization. In addition, these studies also revealed that the C-terminal region of Gli proteins is important for their ciliary localization [17, 45].

The PY-NLS/Kapβ2 nuclear import system has been implicated in the ciliary targeting of several other proteins, including Kif17 and RP2 protein [16, 20]. In addition, depletion of importinβ2/Kapβ2 inhibited ciliary localization of RP2 [20]. Hence, the PY-NLS/Kapβ2 system is likely to play a broad role in the ciliary targeting of nonmembrane proteins. By contrast, Kapβ2 depletion did not affect ciliary localization of Smo (S3 Fig), suggesting that Smo ciliary localization is regulated by a distinct mechanism. Indeed, Smo ciliary localization is regulated by a CLS that is different from the PY-NLS [10], as well as by a Septin family protein, Septin2, localized at the base of the ciliary membrane [46]. In addition, Smo ciliary localization is regulated by phosphorylation and sumoylation of its C-terminal intracellular tail [14, 47], as well as by its association with β-arrestin and BBSome [48, 49].

The observations that a nuclear transport system is involved in ciliary targeting and many nucleoporins (Nups) are localized at ciliary base and that the ciliary base has a diffusion barrier similar to that of nuclear pores led to the proposal that the ciliary base may contain a nuclear pore-like structure [50–52]. However, other studies revealed that the ciliary diffusion barrier is mechanistically distinct from those of the nuclear pore complex [53, 54]. Furthermore, a recent study using super-resolution imaging revealed that Nup188 forms 2 barrel-like structures with dimensions and organization incompatible with a nuclear pore complex (NPC)-like ring, arguing against Nups forming a ciliary pore complex at ciliary bases [55, 56]. Therefore, it is possible that Nubs form a different type of diffusion barrier at ciliary bases, which could explain why canonical NLS does not function as a ciliary targeting signal. It is also possible that the ciliary bases may contain additional diffusion barriers that need to be overcome through other mechanisms. In this regard, it is important to note that the C-terminal half of Gli2 also contains a sequence essential for its ciliary localization (Fig 3) [17, 23]. It is possible that this sequence may bind a factor or factors that assist Gli proteins in crossing the diffusion barrier at the ciliary base. Alternatively, it may bind a motor protein that actively transports Gli proteins to the ciliary base. Indeed, a previous study revealed that a cytoplasmic microtubule network is required for ciliary targeting of Gli2 [21]. Furthermore, fusing the CLS from Kif17 to a nonciliary kinesin, the Kinesin-1 subunit kinesin heavy chain (KHC), resulted in the ciliary localization of KHC, whereas deletion of the motor domain from Kif17 resulted in the nuclear localization of the truncated Kif17, suggesting that motor domain may act in conjunction with CLS to target Kif17 for ciliary localization [16]. A recent study revealed that the heterotrimeric kinesin-2 complex containing Kif3A/Kif3B/KAP3 interacts with and regulates Gli protein function; however, this motor complex binds Gli2 and Gli3 through the N-terminal but not the C-terminal region of the Gli proteins [57]. Identification and characterization of protein(s) interacting with the C-terminal region of Gli2 will provide further insight into the ciliary targeting mechanism for Gli proteins.

Our study also provides new insight into the role of ciliary localization of Gli in the regulation of Gli activity. Disrupting primary cilia affected the formation of both Gli^R^ and Gli^A^, leading to ectopic but low levels of Hh signaling activity [12, 22]. It has been shown that protein kinase A (PKA) holoenzyme, as well as proteasome, are enriched at the ciliary base [58, 59]. In the resting state, a cilium-localized GPCR, Gpr161, increases the local production of cAMP and thus PKA catalytic activity at the ciliary base, which is essential for Gli processing [60]. Upon Hh stimulation, Gpr161 moves out of the cilia, leading to reduced local production of cAMP and PKA activity, and subsequent inhibition of Gli processing [60]. Similarly, disrupting the primary cilia may abolish the local production of cAMP and PKA catalytic activity, resulting in inhibition of Gli processing. Furthermore, inhibition of PKA activity within cilia using a cilium-tethered PKA inhibitor also impaired Gli processing, leading to the proposal that Gli proteins need to enter the cilia in order to be phosphorylated and processed [61]. Here, we showed that compromised ciliary localization of Gli2 and Gli3 due to Kapβ2 knockdown did not significantly affect their proteolytic processing into Gli^R^ (Fig 7E and 7E′). It is possible that residual ciliary localization of Gli2/3 may account for their normal processing. Alternatively, Gli2/3 can be phosphorylated and processed at the ciliary base in response to a local gradient of PKA activity. By contrast, ciliary localization of Gli proteins is critical for Gli^A^ formation in response to Hh. In Kapβ2-depleted cells, both phosphorylation and nuclear import of Gli2^FL^ were compromised, and more Gli2 ^FL^ was bound by Sufu in the nucleus (Fig 7F–7H), which could explain the diminished Gli^A^ activity (Fig 4C–4E). We propose that ciliary localization of Gli2 in the presence of Hh allows it to be modified (phosphorylated, for example) and converted into Gli^A^ that can escape the inhibition imposed by Sufu. In *Sufu-/-* cells, however, Gli2 is constitutively active and its ciliary localization is no longer required for its activation, which explains why Kapβ2 depletion has no effect on Gli^A^ in *Sufu-/-* MEFs (Fig 4F).

Aberrant Hh pathway activity has been implicated in many types of cancer, including BCC and medulloblastoma, and small molecule Smo inhibitors have been used to treat Hh-driven cancers [62]. However, patients treated with Smo inhibitors often acquired resistance due to mutations that block drug binding [63, 64]. Our finding that Kapβ2 depletion affects Hh signaling downstream of Smo makes it a potential therapeutic target for the treatment of Smo-inhibitor–resistant cancers.

## Materials and methods

### DNA constructs

Wild-type mouse Gli2/3 and their mutants (mPY, mNLS, and mPY+mNLS), as well as mGli2-Ci chimera proteins (GliNCiC, CiNGliC and CiNGliC^mPY^), are tagged with 6 copies of Myc epitope at their N-termini and subcloned into the *pcDNA3*.*1(+)* vector, digested with *EcoRI* and *XhoI*. For lentiviral protein-expressing constructs, N-terminally 6XMyc-tagged mouse Gli2 (wild type [WT] and mPY) and mouse Smo; C-terminally Flag-tagged mouse Sufu; and human Kapβ2 were cloned into the *FUXW* vector digested with *XbaI* and *BamHI*. Flag-lacZ (FZ) and FZ-Gli2PY were described previously [19]. Flag-Gli1 construct was described previously [13]. All the constructs were made by using Gibson Assembly Master Mix (NEB E2611S).

### Cell culture, transfection, immunoprecipitation, immunostaining, and western blot analysis

NIH3T3 cells were cultured in DMEM containing 10% Bovine Calf Serum (ATCC). HEK 393T and *Sufu -/-* MEF cells were cultured in DMEM with 10% Fetal Bovine Serum (FBS) (Sigma Aldrich). Shh treatment was done by serum starvation for 24 hours (0.5% Bovine Calf Serum), then adding a recombinant mouse Shh N-terminal fragment (R&D Systems #464-SH) at 1 ug/ml overnight. Smoothened agonist SAG (Sigma Aldrich) treatment was done at 200 ng/ml for 8–12 hours. Cell transfections were performed using PolyJet in vitro DNA Transfection Reagent (SignaGen) following manufacturer’s instruction. Immunoprecipitation, immunostaining, and western blot analyses were carried out as described previously [47]. The antibodies used in this study are listed as follows: anti-Myc (9E10, Santa Cruz Biotechnology), anti-β-galactosidase (A11132, Life Technologies), anti-acetylated tubulin (T7451, Sigma Aldrich), anti-mGli2 (AF3635, R&D Systems), anti-mGli3 (AF3690, R&D Systems), anti-mKapβ2 (Ab10303, Abcam), anti-α-tubulin (T9026, Sigma Aldrich), anti-Histone3 (Ab1791, Abcam), and anti-BrdU (B8434, Sigma Aldrich).

### Luciferase assay and RT-qPCR

*8XGliBS* luciferase (*Gli-luc*) assay was performed using Dual Luciferase Reporter Assay System (Promega) and FLUOstar OPTIMA (BMGLABTCH). Cells were seeded in 6-well plates and transfected with *8XGliBS* reporter and *pRL-TK* at a 4:1 ratio (as well as other plasmids, if necessary). The day after transfection, cells were treated with Shh or SAG, subjective to lysis and determined for luciferase activity. For quantitative reverse transcription PCR (RT-qPCR) with cell samples, total RNA was extracted from cell using RNeasy Plus Mini Kit (Qiagen), cDNA was synthesized with iScript cDNA synthesis kit (Bio-rad), and qPCR was performed using iQ SYBR Green System (Bio-rad) and a Bio-rad CFX96 real-time PCR system. Glyceraldehyde 3-phosphate dehydrogenase (GADPH) expression level was used a normalization control. The primer pairs used were as follows:

GAPDH, GTGGTGAAGCAGGCATCTGA(F) and GCCATGTAGGCCATGAGGTC(R)

Gli1, GTGCACGTTTGAAGGCTGTC(F) and GAGTGGGTCCGATTCTGGTG(R)

Gli2, AGCTCCACACACCCGCAACA(F) and TGCAGCTGGCTCAGCATCGT(R)

mKapβ2, ATCTTGGTCTTGGGTTCTCTG(F) and CCTTCAGCATGTTCCATTTCTG(R)

Ptch1, GAAGCCACAGAAAACCCTGTC(F) and GCCGCAAGCCTTCTCTAGG(R)

CyclinD1, AGACCTGTGCGCCCTCCGTA(F) and CAGCTGCAGGCGGCTCTTCT(R)

N-Myc, GTCTTCCCCTTCCCGGTGAAC(F) and CAAGGTATCCTCTCCGGAGGTGC(R)

For RT-qPCR with zebrafish samples, about 50 zebrafish embryos at 24 hours post-fixation (hpf) were lysed to extract the RNA by TRIzol (Invitrogen) following the standard protocol. 1 μg of RNA was used for reverse transcription by ReverTra Ace qPCR RT Master Mix with gDNA Remover (TOYOBO). Real-time PCR was performed on ABI Fast7500 with Maxima SYBR Green qPCR Master Mix (Thermo Fisher Scientific). The primer pairs used were as follows:

mKapβ2, GAACGCAAGCCCTAATGCTG(F) and GCATATGTGGAAGGAGACGG(R)

fkd4, GCTTCACTGAACCATTTCGCA(F) and CTGAGCCATAATACATCTCGCTG(R)

hhip, CTTACGAGCCAAGTGTGAACTG(F) and TGCTGTCTTTCTCACCGTCC(R)

Gli1, TTCTTGGTTTACTTGAAGGCAGAG(F) and GCTCATTATTGATGTGATGCACC(R)

nkx2.2b, CAAATATCCAGTGCCGTCAGC(F) and CGCTCTAACTCAAAGGTTTGAGTC(R)

ptch2, TCCTCCTTATGAGTCCCAAACAG(F) and CATGAACAACCTCAACAAACTTCC(R)

axin2, CTTAAACCTGCCACTAAGACCT(F) and CATTCTCCTCCATAGCCGTC(R)

GAPDH, CATCACAGCAACACAGAAGACC(F) and ACCAGTAAGCTTGCCATTGAG(R)

### Lentivirus production

HEK293T cells were seeded and transfected with *psPAX2* and *VSVG*, along with *pLKO*.*1-puro* (for shRNA lentivirus) or *FUXW* (for protein expressing lentivirus). After 48 hours, virus-containing culture media was collected, filtered, and centrifuged at 20,000 g for 2 hours; the resultant precipitant was resuspended in a small volume of culture medium and stored at −80°C for future use. Mission shRNA plasmids against eGFP (control), mGli2 (TRCN0000219066), and mKapβ2 (TRCN0000295632 and TRCN0000295586) were purchased from Sigma Aldrich. The mGli1 shRNA plasmid was a kind gift from Dr. Jiang Wu’s lab. Myc-tagged WT and PY mutant mGli2, mSmo, Flag tagged human Kapβ2 were subcloned into *FUXW* vector under the control of a ubiquitin promoter.

### Chromatin immunoprecipitation

NIH3T3 cells treated with vehicle or SAG were crosslinked with paraformaldehyde for 15 minutes at room temperature with agitation. After quenching, cells were lysed, sonicated, centrifuged, and immunoprecipitated with anti-Myc antibody. Precipitated DNA was purified and subjective to real-time PCR. The primer pairs used were as follows:

S1, CTTCCAAGACCCGGGTTTCTC(F) and AATAATGGTAATGAAGAGAG(R)

S2, CCCTCTGAACCATTTTCCCAG(F) and AGCTTTCATTGTAGAGTAGAG(R)

S3, CGAGACAGGATTTCTCTGTGT(F) and TTTAGGCAGGGCATGGTGGCG(R)

C1, GAGGAAGGTTTACATTAAATTG(F) and GAGAAACTTTGTCTCTATCA(R)

### Primary CGNP, medulloblastoma cell culture

Primary CGNP cells were dissociated from P3–P4 mice and cultured in DMEM/F12 medium containing 25 mM KCl, N2 supplement (Invitrogen), and 10% FBS (Sigma Aldrich). Shh-conditioned media derived from Shh-CM 293T cells was added in the above culture medium at a 1:20 ratio [35]. For primary medulloblastoma cell culture, tumor cells from SmoM2, CAG-creER mice [37, 40] with spontaneously occurring medulloblastoma were dissociated and cultured in the same medium as above except for Shh-conditioned media. Corresponding lentiviruses were added to the culture medium for the above 2 primary cells immediately after seeding and were maintained for 3 days. BrdU was applied 2 hours before immunostaining, and viable cell number was determined by CellTiter-Glo Luminescent Cell Viability Assay Kit (Promega).

### Ethics statement

All experimental procedures were approved by the local IACUC animal research committee (University of Texas Southwestern Medical Center, protocol: APN2009-0018) and complied with NIH guidelines (PHS Animal Welfare Assurance D16-00296 [A3472-01]). Extra care was taken of animals that suffered from medulloblastoma.

### MO knockdown, in situ hybridization, and immunostaining of zebrafish embryos

Antisense MOs (Gene Tools) were microinjected into 1-cell stage embryos according to the standard protocols. A 4-nl volume of mixed MOs was injected at the concentration of 0.075 mM Kapβ2-MO1 and either 0.15 mM Kapβ2-MO2 or 0.15 mM Smo MO. MO sequences used were Kapβ2-MO1(5′-CCATGCTGTATCGGGCTTCTCTTAC-3′), Kapβ2-MO2(5′-TCGGGTTTCCACTGGCACTCCATC-3′), Smo-MO(5′-CTGGGCAGATGAGACTGGATGATTA-3′), and standard control MO.

Zebrafish embryos of the AB strain were obtained from the Zebrafish Core Facility at the Shanghai Institute of Biochemistry and Cell Biology. Whole mount in situ hybridization of zebrafish embryos was performed according to standard protocols [65]. Immunostaining of zebrafish embryos was performed as previously described [66]. In brief, Zebrafish embryos were fixed for 3 hours at room temperature in 4% formaldehyde and stored in methanol at −20°C overnight. Staining was performed in PBStwA (PBStw + 1% BSA) using anti-Engrailed (Developmental Studies Hybridoma Bank) and F59 anti-Myosin heavy chain antibodies (Santa Cruz). Images of zebrafish embryos were acquired under a confocal microscope (LAS SP5) using a 20×/0.7 NA objective (Leica) at room temperature.

## Supporting information

S1 Fig The PY-NLS is not sufficient for Gli ciliary localization. (**A**) Diagram of Flag-tagged with or without Gli3 PY-NLS fused to its C-terminus. (**B**) Subcellular localization of FZ and FZ-Gli2PY expressed in NIH3T3 cells. and immunostained with LacZ and acetylated tubulin (primary cilium) antibodies.
(TIFF)

S2 Fig Kapβ2 inactivation affects ciliary localization of Gli1 and Gli3. (**A**) Knock-down efficiency by mKapβ2 shRNA1 and shRNA2 in NIH3T3 cells. (**B**) Quantitation of mGli2 positive cilia in NIH3T3 cell infected with lentiviruses expressing GFP shRNA (Ctr), mKapβ2 shRNA1 or mKapβ2 shRNA2 and with or without ShhN treatment. (**C-F**) Ciliary localization of Myc-Gli3 (**C-D**) or Flag-Gli1 (**E-F**) transfected into control or mKapβ2 depleted NIH3T3 cells with or without hKapβ2 coexpression. Data are means ± SD from two independent experiments (100 cells were counted each condition). ** P<0.01, NS not significant. The underlying data for this figure can be found in S1 Data.
(TIFF)

S3 Fig Kapβ2 is not required for ciliary localization of Smo. (**A-B**) Ciliary localization of Myc-tagged mSmo transfected into control or mKapβ2 depleted NIH3T3 cells treated with or without Shh. Data are means ± SD from two independent experiments (100 cells were counted each condition). (C) Kif7-YFP was localized to primary cilia in 100% of both control and mKapβ2 depleted NIH3T3 cells (N = 50 cells for each genotype). The underlying data for this figure can be found in S1 Data.
(TIFF)

S4 Fig Kapβ2 is a Shh responsive gene whose expression is upregulated in Shh subgroup of medulloblastoma. (**A**) mKapβ2 mRNA level in medulloblastoma samples (n = 3) and adjacent wild type cerebellar tissues (n = 2) from *CAGGS-CreER*; *R26-SmoM2* mice (GDS3008). (**B**) Kapβ2 and Kapβ1 mRNA levels in Shh (n = 10) and Wnt (n = 8) subtype human medulloblastoma samples (GDS4471). Data are means ± SD. *** P< 0.001, NS: not significant. Kapβ2 but not Kapβ1 was upregulated in Shh subgroup of medulloblastoma samples. (**C**) Relative mRNA levels of the indicated genes from 24 hpf zebrafish embryos injected with control or Smo MOs were measured by RT-qPCR. Data are means ± SD from three independent experiments. ** P<0.01, *** P< 0.001, NS: not significant. The underlying data for this figure can be found in S1 Data.
(TIFF)

S1 Data Raw data. (XLSX)

## Abbreviations

aa: amino acid
BCC: basal cell carcinoma
BrdU: bromodeoxyuridine
CGNP: cerebellum granule neuron precursor
CHIP: chromatin immunoprecipitation
Ci: Cubitus interruptus
CiN: Ci1-440
CLS: ciliary localization signal
EGL: external granule layer
FZ: flag-lacZ
GFP: green fluorescent protein
GLI^A^: Gli activator
GPCR: G-protein-coupled receptor
Hh: Hedgehog
hKapβ2: human karyopherinβ2
hpf: hours post-fixation
Kapβ2: karyopherinβ2
KIF: kinesin family member
KHC: kinesin heavy chain
MEF: mouse embryonic fibroblast
mKapβ2: mouse karyopherinβ2
MO: morpholino
NLS: nuclear localization signal
NPC: nuclear pore complex
Nup: nucleoporin
PKA: protein kinase A
Ptc: Patched
PY-NLS: PY-type nuclear localization signal
RNAi: RNA interference
RP2: retinitis pigmentosa 2
RT-qPCR: quantitative reverse transcription PCR
Shh: sonic hedgehog
Shh-N: sonic hedgehog N-terminal fragment
shRNA: short hairpin RNA
Smo: Smoothened
WT: wild type
YFP: yellow fluorescent protein
