# Supplementary figures and images for: Regulation of Gli ciliary localization and Hedgehog signaling by the PY-NLS/karyopherin-β2 nuclear import system
Source: PLoS Biol. 2017 Aug 4;15(8):e2002063. doi: 10.1371/journal.pbio.2002063 (PMC5544186; doi:10.1371/journal.pbio.2002063)

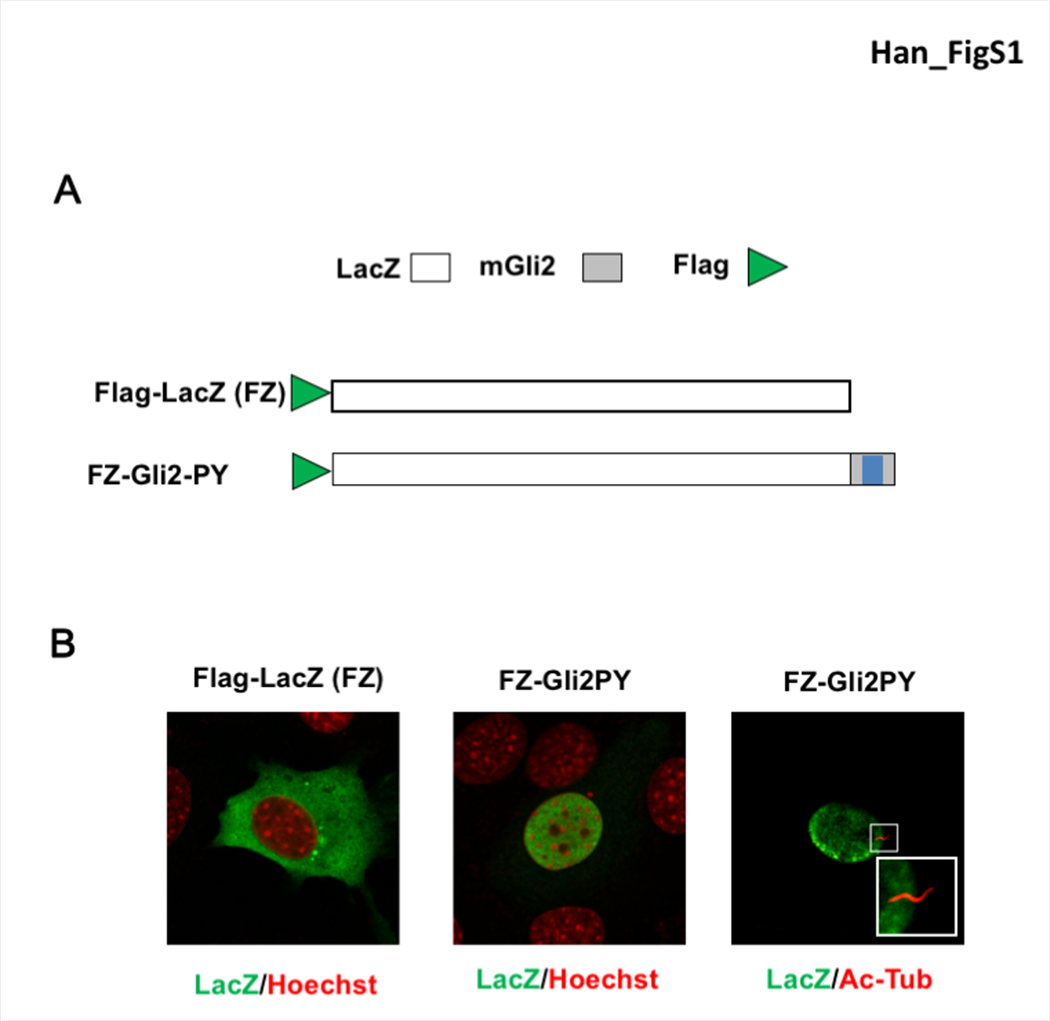

Supplement: S1 Fig — (A) Diagram of Flag-tagged with or without Gli3 PY-NLS fused to its C-terminus. (B) Subcellular localization of FZ and FZ-Gli2PY expressed in NIH3T3 cells. and immunostained with LacZ and acetylated tubulin (primary cilium) antibodies. (TIFF) [file pbio.2002063.s001.tiff]

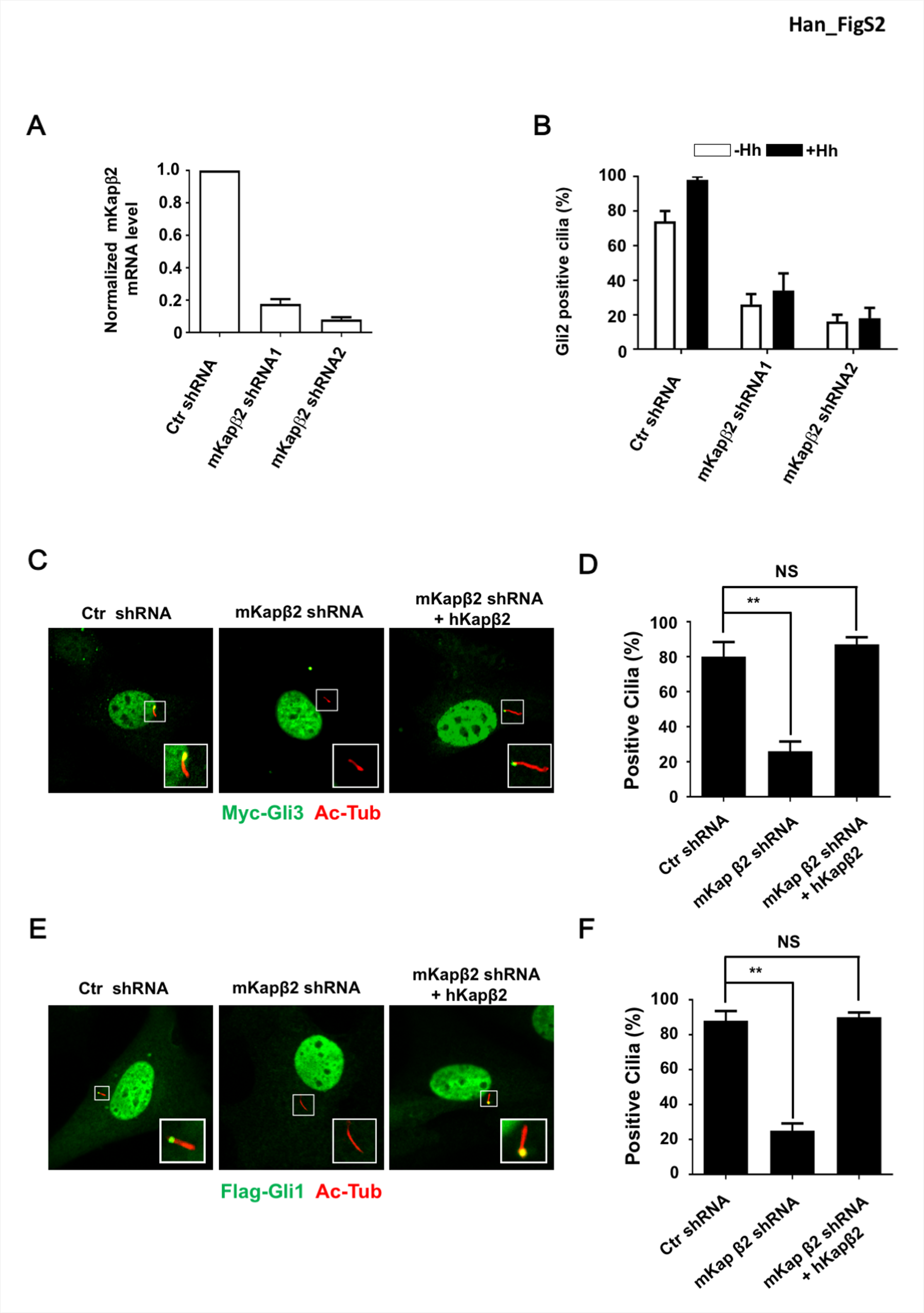

Supplement: S2 Fig — (A) Knock-down efficiency by mKapβ2 shRNA1 and shRNA2 in NIH3T3 cells. (B) Quantitation of mGli2 positive cilia in NIH3T3 cell infected with lentiviruses expressing GFP shRNA (Ctr), mKapβ2 shRNA1 or mKapβ2 shRNA2 and with or without ShhN treatment. (C-F) Ciliary localization of Myc-Gli3 (C-D) or Flag-Gli1 (E-F) transfected into control or mKapβ2 depleted NIH3T3 cells with or without hKapβ2 coexpression. Data are means ± SD from two independent experiments (100 cells were counted each condition). ** P<0.01, NS not significant. The underlying data for this figure can be found in S1 Data. (TIFF) [file pbio.2002063.s002.tiff]

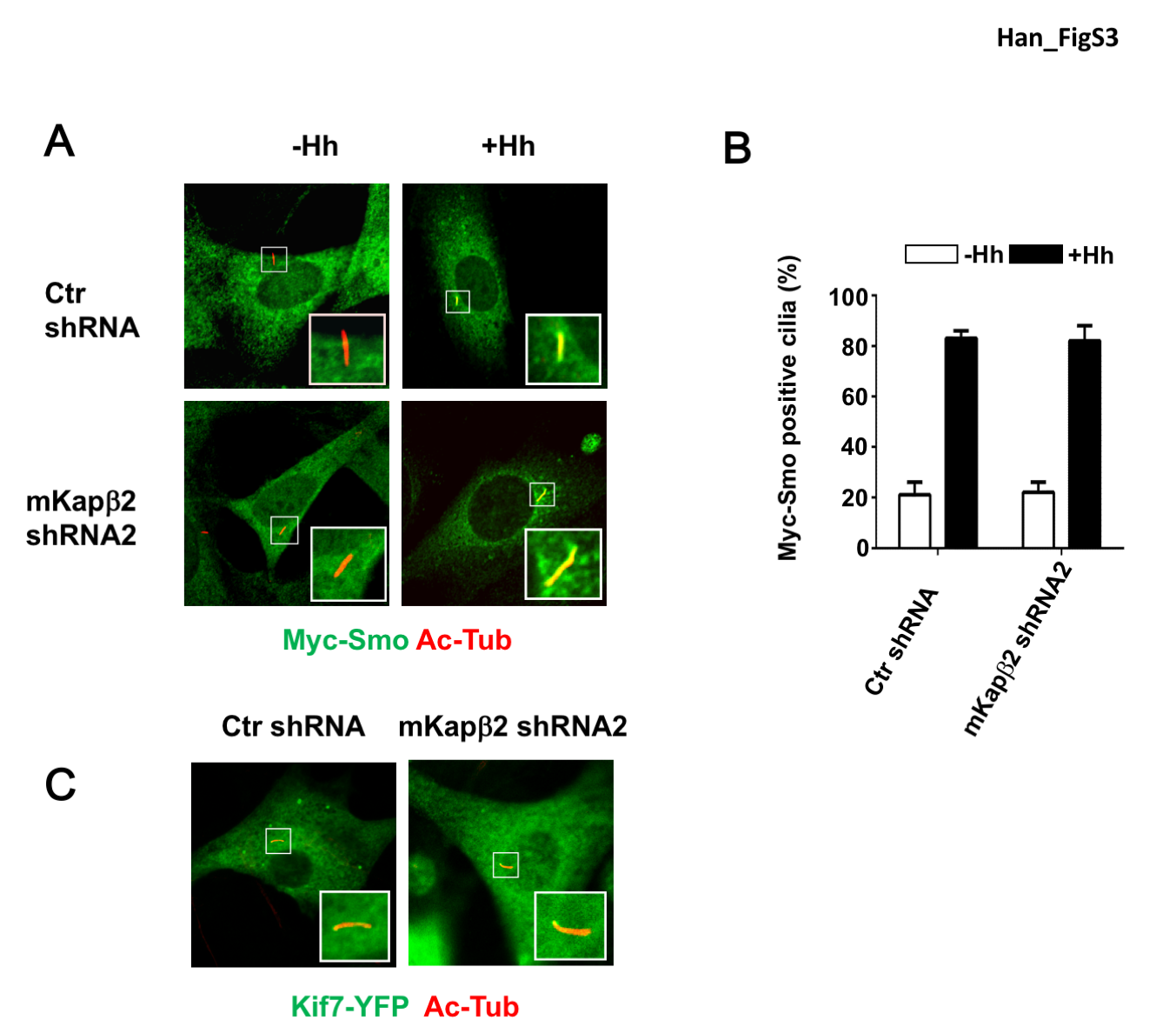

Supplement: S3 Fig — (A-B) Ciliary localization of Myc-tagged mSmo transfected into control or mKapβ2 depleted NIH3T3 cells treated with or without Shh. Data are means ± SD from two independent experiments (100 cells were counted each condition). (C) Kif7-YFP was localized to primary cilia in 100% of both control and mKapβ2 depleted NIH3T3 cells (N = 50 cells for each genotype). The underlying data for this figure can be found in S1 Data. (TIFF) [file pbio.2002063.s003.tiff]

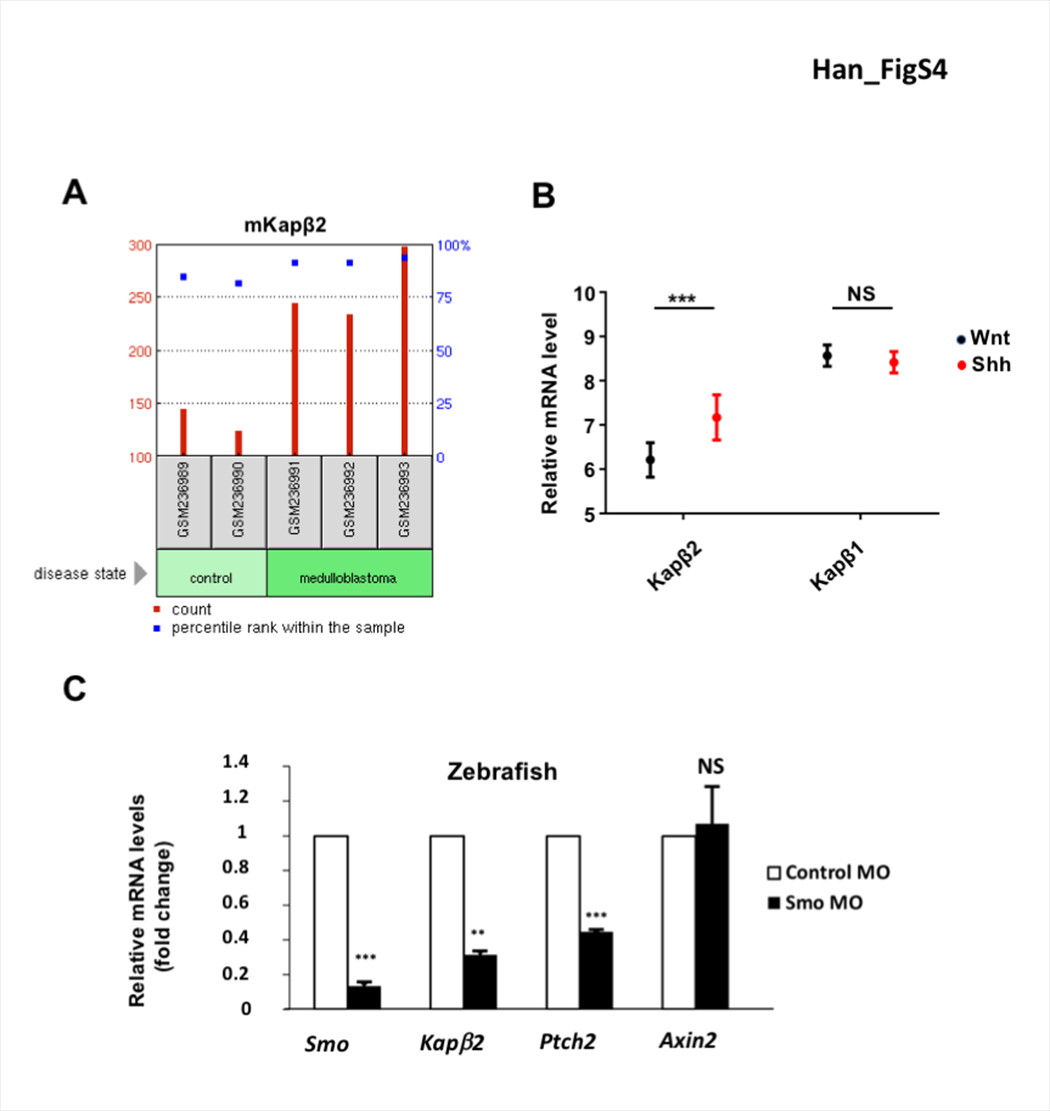

Supplement: S4 Fig — (A) mKapβ2 mRNA level in medulloblastoma samples (n = 3) and adjacent wild type cerebellar tissues (n = 2) from CAGGS-CreER; R26-SmoM2 mice (GDS3008). (B) Kapβ2 and Kapβ1 mRNA levels in Shh (n = 10) and Wnt (n = 8) subtype human medulloblastoma samples (GDS4471). Data are means ± SD. *** P< 0.001, NS: not significant. Kapβ2 but not Kapβ1 was upregulated in Shh subgroup of medulloblastoma samples. (C) Relative mRNA levels of the indicated genes from 24 hpf zebrafish embryos injected with control or Smo MOs were measured by RT-qPCR. Data are means ± SD from three independent experiments. ** P<0.01, *** P< 0.001, NS: not significant. The underlying data for this figure can be found in S1 Data. (TIFF) [file pbio.2002063.s004.tiff]
